# Multiscale Modeling of Metabolism and Macromolecular Synthesis in *E. coli* and Its Application to the Evolution of Codon Usage

**DOI:** 10.1371/journal.pone.0045635

**Published:** 2012-09-28

**Authors:** Ines Thiele, Ronan M. T. Fleming, Richard Que, Aarash Bordbar, Dinh Diep, Bernhard O. Palsson

**Affiliations:** 1 Center for Systems Biology, University of Iceland, Reykjavik, Iceland; 2 Faculty of Industrial Engineering, Mechanical Engineering and Computer Science, University of Iceland, Reykjavik, Iceland; 3 Department of Bioengineering, University of California San Diego, La Jolla, California, United States of America; 4 Department of Biochemistry and Molecular Biology, Faculty of Medicine, University of Iceland, Reykjavik, Iceland; Tel Aviv University, Israel

## Abstract

Biological systems are inherently hierarchal and multiscale in time and space. A major challenge of systems biology is to describe biological systems as a computational model, which can be used to derive novel hypothesis and drive experiments leading to new knowledge. The constraint-based reconstruction and analysis approach has been successfully applied to metabolism and to the macromolecular synthesis machinery assembly. Here, we present the first integrated stoichiometric multiscale model of metabolism and macromolecular synthesis for *Escherichia coli* K12 MG1655, which describes the sequence-specific synthesis and function of almost 2000 gene products at molecular detail. We added linear constraints, which couple enzyme synthesis and catalysis reactions. Comparison with experimental data showed improvement of growth phenotype prediction with the multiscale model over *E. coli*’s metabolic model alone. Many of the genes covered by this integrated model are well conserved across enterobacters and other, less related bacteria. We addressed the question of whether the bias in synonymous codon usage could affect the growth phenotype and environmental niches that an organism can occupy. We created two classes of *in silico* strains, one with more biased codon usage and one with more equilibrated codon usage than the wildtype. The reduced growth phenotype in biased strains was caused by tRNA supply shortage, indicating that expansion of tRNA gene content or tRNA codon recognition allow *E. coli* to respond to changes in codon usage bias. Our analysis suggests that in order to maximize growth and to adapt to new environmental niches, codon usage and tRNA content must co-evolve. These results provide further evidence for the mutation-selection-drift balance theory of codon usage bias. This integrated multiscale reconstruction successfully demonstrates that the constraint-based modeling approach is well suited to whole-cell modeling endeavors.

## Introduction

Cell-scale modeling is one of the great goals of computational biology. In fact, in 2002 an international *Escherichia coli* alliance was formed with the aim to generate data and tools necessary to formulate a whole cell computer representation of this bacterium [1]. Many computational modeling techniques exist, differing in underlying assumptions, captured complexity, and key properties of the modeled systems that are described. In the constraint-based reconstruction and analysis (COBRA) approach, biochemical transformations are described based on reaction stoichiometric and physico-chemical properties obtained from genome annotation, biochemical, and physiological data [2]. Biochemical reactions networks are reconstructed in a bottom-up manner and serve as knowledge-bases as they summarize existing knowledge about cellular pathways in a target organism in a well structured, mathematical manner. The reconstruction process has been described in detail in a 96-step standard operating procedure [3], which was the basis for a semi-automated, web-based reconstruction tool [4] that permits the rapid creation of curated draft metabolic reconstructions for prokaryotes. Metabolic reconstructions have been published for a large number of prokaryotes, such as biotechnological relevant [5]–[7] and biomedical interesting bacteria [8]–[12], as well as for numerous eukaryotes [13]–[17]. These reconstructions can be converted into condition-specific, predictive models [2], [3] and their properties can be interrogated using different mathematical tools [18], many of which are based on linear programming, which is well suited for large-scale modeling. The COBRA approach together with manually curated, genome-scale metabolic reconstructions has been successfully employed for many biotechnological and biomedical applications [19], [20].

The metabolic reconstruction of *E. coli* has been updated, refined, and extended over the last two decades [21], [22]. In this study, we employed a recent, very comprehensive version of the metabolic reconstruction, *i*AF1260, which accounts for function of 1260 metabolic genes and represents almost 30% of the open reading frames (ORF) in *E. coli*’s genome [5]. We recently reconstructed the first genome-scale, stoichiometric network of the macromolecular synthesis machinery of *E. coli* [23]. It accounts for 303 gene products, including ribosomal proteins, RNA polymerase, tRNA, and rRNA. It represents the synthesis and assembly of all known functional components involved in macromolecular synthesis. Here, we integrate these two reconstructions into a Metabolic-Expression (‘ME’) matrix reconstruction that accounts for the synthesis of almost 2,000 *E. coli* genes. To-date, only few examples of integrated networks of cellular functions have been published, including i) a metabolic-regulatory network using metabolic reconstruction and transcriptional regulatory network in form of Boolean expressions, for *E. coli* [24]; and ii) metabolic-signaling-regulatory models [25], [26]. However, these integrated functional networks do not explicitly account for proteins (enzymes and regulators) and they employ other modeling tools than COBRA (e.g., ordinary differential equations or Boolean logic).

The degeneracy of the genetic code implies that one or more cognate tRNA species can recognize the same codon (a triplet of nucleotides using a four letter code) on a messenger RNA (mRNA), while a tRNA species can also read two or more synonymous codons (Figure S1). There is a unique set of codons and tRNA species per amino acid. The number of amino acids and codons is fixed to 20 and 64, respectively, but the number of tRNA genes varies widely (29–126) even between closely related organisms. Fast growing bacteria contain a higher number of tRNA genes for a smaller set of the possible anticodons (corresponding triplets on the tRNA species) [27]. At the same time, the frequency of synonymous codon use differs between organisms, within genomes, and along genes, a phenomenon known as codon usage bias.

So far, key questions of molecular evolution of genome sequences could not be investigated with COBRA networks as they do not explicitly account for genes and proteins in a sequence-specific manner. In this study, we developed a novel framework that permits the analysis of sequence-related questions and potential phenotypic consequences.

## Results

In this study, we present a comprehensive, mechanistically detailed, integrated network of metabolism and macromolecular synthesis machinery for *E. coli*, deemed ME-matrix for *m*etabolism and *e*xpression. We first reconstructed and validated this comprehensive, sequence-specific ME-matrix. Then, we determined how conserved the ME-matrix genes are in other bacteria. Lastly, we modified the codon usage of the wildtype model towards more biased and more equilibrated codon usage model strains. Subsequently, we tested the growth phenotypes of the model strains in different environmental conditions to assess the impact of the genotypes (being codon usage pattern) on the growth phenotypes.

### An Integrated Reconstruction of Metabolism and Macromolecular Synthesis Machinery

#### Creation of the integrated reconstruction

**Figure 1:**
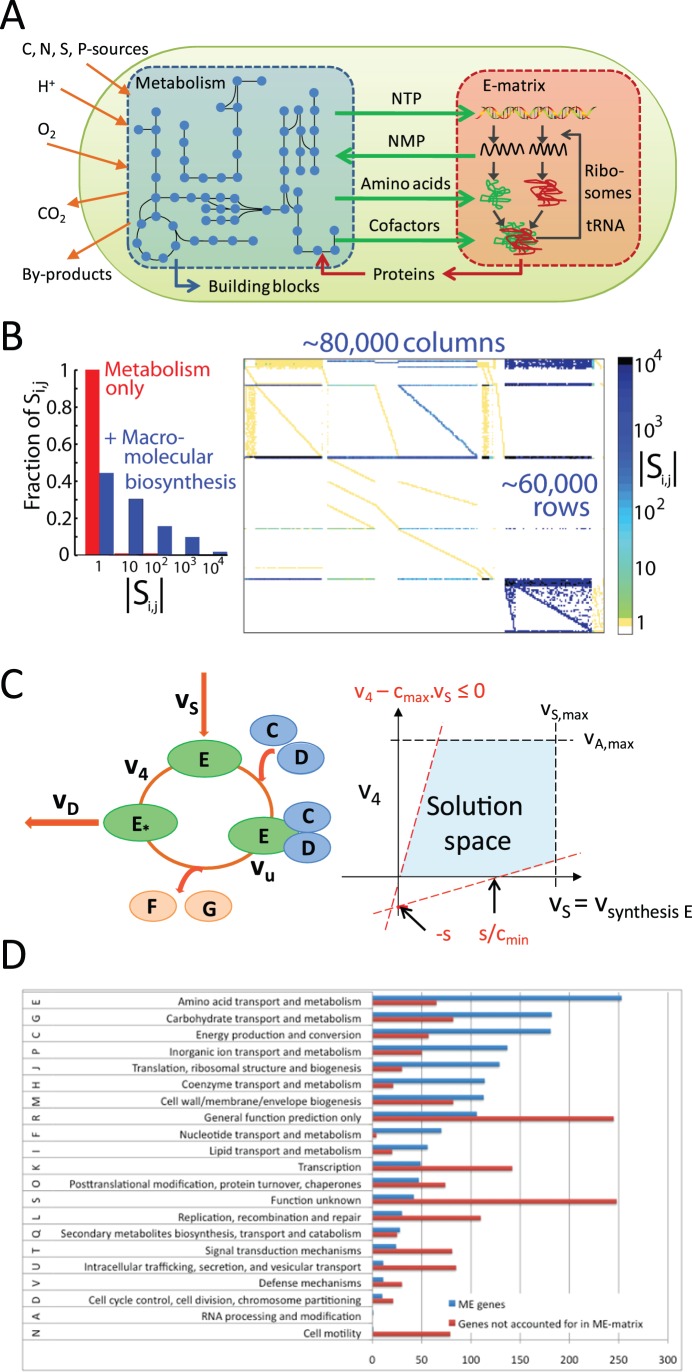
Overview of the ME-matrix. **A:** Functional synergy between metabolism and macromolecular synthesis. **B:** ME-matrix histogram of stoichiometric coefficients (left) and sparsity pattern (right). The stoichiometric coefficients are spread over four orders of magnitude because of the difference in biochemical moieties required for metabolic and macromolecular synthesis reactions. **C:** Coupling constraints were be added to the ME-matrix to link (or “couple”) the flux through a biosynthetic flux, 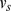, (e.g., transcription) with the corresponding utilization reaction(s), 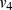, (e.g., translation) [28]. 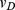 = protein dilution. **D:** Distribution of clusters of orthologous groups is shown for a total of 2,806 *E. coli* genes, of which 1,436 are in the ME-matrix.

We assembled an integrated stoichiometric reconstruction of *E. coli* MG1655’s metabolic (M-matrix) [5] and macromolecular synthesis machinery (E-matrix) [23] networks (Figure 1A). We added transcription and translation reactions for all metabolic genes in the M-matrix to the E-matrix. The metabolic reactions were then reformulated to include the catalyzing enzymes as reactants. The ME-matrix generation involved adding enzymes, enzyme complexes, and inactive enzymes to each metabolic reaction (see Materials and Methods section for details). Functional overlap between the M-matrix and the E-matrix exists on two points: i) exchange reactions of the E-matrix and the metabolic synthesis reactions; and ii) the metabolites incorporated by the E-matrix into RNA and proteins that are also consumed by the biomass reaction of the metabolic network (Figure 1A).

#### Addition of coupling constraints

The conversion of a reconstruction into a mathematical model normally consists of the definition of systems boundaries, the addition of exchange and demand reactions, and the application of condition-specific constraints on exchange and/or intracellular reactions [3]. To convert the ME- matrix reconstruction into condition-specific ME-matrix models, three sets of constraints were applied, i) constraints on the exchange reactions to simulate different environmental conditions, ii) constraints on the maximal transcription rate for stable and messenger RNA, and iii) coupling constraints. The latter linearly constrain the ratio between the flux through a biosynthetic reaction, 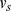 (e.g., transcription), and the flux through the corresponding utilization reaction(s), 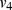 (e.g., translation), (Figure 1C). Coupling constraints are a linear approximation to the non-linear relationship between the synthesis of a macromolecule and its utilization. The formulation of the constraints ensure that when a biosynthetic flux is zero then its utilization flux is also zero. An upper bound on the coupling constraint ensures that a higher rate of utilization also leads to a higher biosynthetic flux. In the ME-matrix, these coupling constraints forced the network to produce more gene products when they were highly used and represents a limit on enzyme capacity (see also Materials and Methods). By applying bounds on the synthesis and utilization ratio rather than fixing it, which would correspond to a straight line in Figure 1C, we permit the model to find the appropriate ratio for a given simulation condition. This resulting ratio can be used to calculate the enzyme turnover rates, for example, in the case of enzyme synthesis and utilization reactions. Conversely, if such turnover rates are known for particular enzymes, one can apply them as additional constraints to the model, thereby, fixing the synthesis and utilization ratio. For interpretation of the coupling constraints refer also to [28].

#### Adjustment of biomass

Metabolic reconstructions generally contain a biomass reaction, which stoichiometrically weights the contribution of metabolic precursors towards synthesis of a new cell [3], [29]. The ME-matrix accounts for the synthesis of almost half of the functions encoded in *E. coli*’s genome. Subsequently, the biomass reaction, which accounts for precursors to the macromolecular building blocks, needed to be adjusted for the fraction of amino acids (AA) and nucleotide triphosphates (NTP) used for synthesis of ME-matrix gene products. We carried out a sensitivity analysis to identify the best parameters, such that the model achieved experimentally observed growth rates (Figure S2). Two main parameters were considered, the fraction of (i) amino acids and (ii) growth associated maintenance (GAM). The latter is included in a biomass reaction to account for the energy necessary to synthesize RNA and proteins (in terms of ATP hydrolysis) [3], [5]. Note that we did not alter the fraction of NTPs since their overall contribution is relatively small in the biomass reaction. We found that a good overlap between *in silico* and *in vivo* growth rate was achieved when the biomass reaction was adjusted to 50% of the amino acid requirement and 50% of the GAM (Figure S2). However, as the composition of the transcriptome and proteome depends on the growth rate and on genetic and environmental conditions, the proposed adjustment to 50% may not always be suitable. In fact, one can employ the measured growth rate at a particular condition to identify the correct percentage of amino acids and GAM required in the biomass reaction. Thus, fine-tuning of these two parameters may lead to an improvement of quantitative growth rate and energy cost predictions, depending on the simulation condition.

#### Content of the ME-matrix

The ME-matrix accounts for 1,260 metabolic genes, 303 macromolecular synthesis machinery genes, and 375 genes without function in the ME-matrix (Table 1). These latter genes were included in the ME-matrix as gene expression is captured in terms of transcription units rather than single genes. Thus, these genes were part of the same transcription units as genes with defined function in the ME-matrix. Overall, these 1938 genes correspond to 1,823 protein coding genes and 115 RNA coding genes captured by the ME-matrix along with their synthesis reactions, at a single nucleotide resolution. The codon usage of ME-matrix is comparable with genomic codon usage. The most frequent codons were CTG (leucine) and GCG (alanine) (Figure S3).

**Table 1:** ME-matrix statistics.

Type	Number
Transcription Units	1,152
Genes	1,937
- Protein coding genes	1,827
- RNA coding genes	110
Network reactions	76,589
Network components	62,212
Coupling constraints between reactions	3,044

Summary of ME-matrix content.

The generation of the synthesis reactions leading to functional gene products has been described in great detail elsewhere [23]. The metabolic reconstruction provided gene-protein-reaction (GPR) associations encoding, via Boolean rules, which gene products catalyze a metabolic function [3]. While GPRs capture heteromeric complexes, they do not contain any information regarding homomers. A total of 252 protein complex formation reactions were added manually based on the GPR association and literature (Table 2). Furthermore, 128 proteins have covalently bound metallo-ions. This information has not been considered in any other biochemical reconstruction. Furthermore, 3548 metabolic units, each consisting of four to seven reactions, depending of the reaction directionality (see Materials and Methods for details), capture the 2042 unique metabolic reactions present in *i*AF1260. The number metabolic unit is higher due to the presence of isozymes, which are captured explicitly in the ME-matrix. In addition, 240 enzyme export reactions from the cytoplasm to the periplasm, and 16 enzyme export reactions from the periplasm to the extracellular space were accounted for. Overall, 26 cellular processes are accurately and sequence-dependent included in the ME-matrix for almost 2000 *E. coli* genes (Table 3). In summary, the ME-matrix reconstruction encompasses many cellular functions detailed in 76,589 reactions and 62,212 components (Figure 1D, Table 1).

**Table 2:** Information used for the synthesis reactions of *E. coli*’s metabolic genes.

Information	Subsystem/Reaction	Source/Reference
Transcription unit	Transcription	EcoCyc [60]
Gene coordinate, direction	Transcription	Riley *et al.* [61]
Gene function	Metabolism	*i*AF1260 [5]
Protein information	Protein complex formation	*i*AF1260 [5], EcoCyc [60], primary literature
Metallo-ion	Metallo-ion binding	EcoCyc [60], protein structure, primary literature
Prosthetic group	Protein complex formation	EcoCyc [60], protein structure, primary literature

**Table 3:** Overview of cellular processes included in the ME-matrix.*^a^*

Subsystem	Cellular process	Number of reactions
RNA metabolism	Transcription	3,561
RNA metabolism	Transcription regulation*^a^*	1,182
RNA metabolism	mRNA degradation	3,646
RNA metabolism	Cleavage of polycistronic mRNA	1,029
RNA metabolism	RNA processing	124
RNA metabolism	rRNA modification	864
RNA metabolism	rRNA formation	38
RNA metabolism	tRNA modification	1,597
Protein metabolism	Translation*^b^*	38,617
Protein metabolism	tRNA charging	177
Protein metabolism	Aminoacyl-tRNA synthetase charging	33
Protein metabolism	Charging EF-Tu	4
Protein metabolism	tRNA activation (EF-TU)	45
Protein metabolism	Protein maturation	3,646
Protein metabolism	Protein folding*^c^*	2,618
Protein metabolism	Metallo-ion binding	128
Protein metabolism	Protein modification	12
Protein metabolism	Protein complex formation	252
Protein metabolism	Protein recycling	1,155
Protein metabolism	Ribosomal assembly	13
Protein metabolism	Ribosomal protein modification	21
Protein metabolism	Iron-sulfur cluster incorporation	6
Iron-sulfur metabolism	Iron-sulfur cluster biosynthesis	6
Cellular metabolism	Metabolism*^d^*	13,819
Others	Demands & Sinks	3,621
Others	Exchange reactions	375
Total number of reactions		76,589

The transcription regulation reactions are currently placeholders for future regulatory information to be added, i.e., that each transcription unit can be active in the ME-matrix without the presence of any transcription regulator (see also [23] for more details).

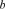
Translation reactions account for one ribosome per mRNA, the maximal possible number of ribosomes per mRNA, i.e., every 17 amino acids [65], and for the half maximal possible number of ribosome per mRNA (see [23] for details).

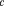
Protein folding accounts for spontaneous, but trigger factor assisted protein folding, for DnaK-dependent folding (based on [66]), and GroEL/ES-dependent folding (based on [67]).

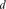
Each metabolic reaction was replaced by at least four reactions (see Material and methods section).

#### Functional Coverage of the ME-matrix

The functional gene coverage included in the ME-matrix reconstruction may be assessed by looking at the distribution of COGs [30] (Figure 1). A total of 2,806 *E. coli*’s genes had an assigned COG function, of which 1,436 were in the ME-matrix reconstruction. The remaining 496 ME-genes had no COG information and thus could not be considered for the functional coverage analysis. The transcription category contains 142 genes that are currently not included in the ME-matrix, as it did not account for transcriptional regulation. Similarly, genes of the replication, cell motility, and signal transduction categories were not captured due to scope limitations of the ME-matrix reconstruction.

### Model Validation

#### Quantitative prediction of growth phenotypes

We compared the growth predictions with experimental data to assess the predictive potential of the ME-matrix. The experimental data were obtained from the literature and correspond to wildtype strains in multiple environmental conditions (i.e., minimal medium supplemented with glucose, glycerol, or lactate in aerobic and anoxic conditions). Furthermore, the wildtype cells have been evolved on minimal medium supplemented with glycerol or lactate and after 60 days of evolution, with optimal growth as the selection pressure, the substrate and oxygen uptake rates have been measured [24], [31]. We compared the ME-matrix predictions with optimal growth rates calculated with *i*AF1260 (Figure 1 A). We found that in many cases the metabolic network predicted too high growth rates, while the ME-matrix growth rates were often below the experimentally measured ones. A main reason for the prediction of lower growth rates was the choice of parameters for the amino acid and GAM contribution remaining in the biomass reaction of the ME-matrix. A sensitivity analysis showed (Figure S2) that these two parameters play a key role in prediction of *in silico* growth rate.

#### Qualitative prediction of growth phenotypes

**Figure 2:**
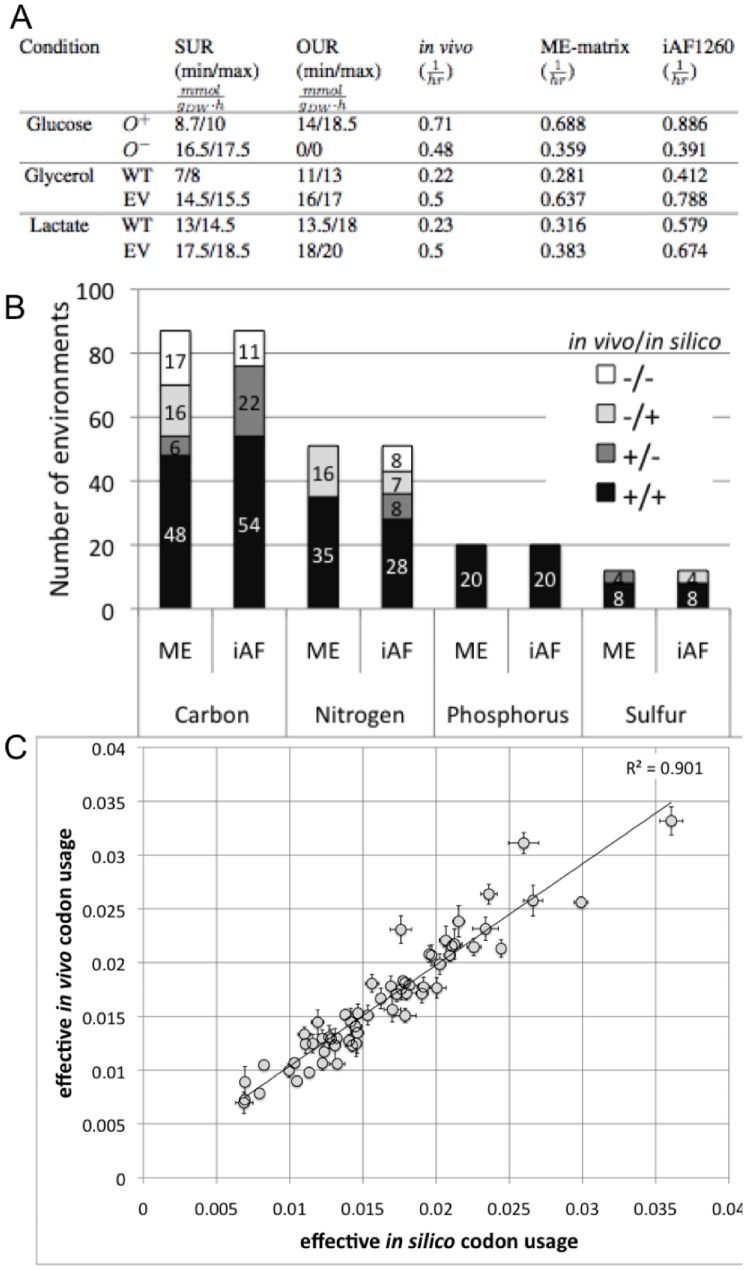
ME-matrix validation. **A:** Comparison of predicted and experimentally determined growth rates [24], [31]. SUR = substrate uptake rate. OUR = oxygen uptake rate. 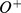 = aerobic. 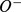 = anaerobic. WT = wildtype. EV = evolved strain. **B:** Comparison of qualitative growth phenotype data with predicted *in silico* growth phenotype of ME-matrix (ME) and of metabolic model (iAF) prediction across 170 environments (from Biolog data). **C:** Correlation between effective *in silico* and *in vivo* codon usage.

We aimed to determine how well the ME-matrix could qualitatively predict growth phenotypes. We compared *in silico* growth phenotypes under 170 different growth conditions with phenotyping data from Biolog (http://www.biolog.com/). The ME-matrix correctly predicted 75% (128/170) of growth phenotypes (Figure 2), whereas *i*AF1260 correctly predicted 76% (129/170) growth phenotypes. The ME-matrix agreed in 85% (144/170) of the predictions with *i*AF1260. It could grow in 16 conditions where *i*AF1260 could not, whereas in 12 conditions, *iAF1260* could grow but the ME-matrix could not. For instance, the ME-matrix, but not *i*AF1260, could grow on 6 carbon sources (decanoate, hexanoate, butyrate, (S)-propane-1,2-diol, 4-aminobutanoate, and glycerol 3-phosphate). In contrast to *i*AF1260, the ME-matrix was unable to grow when formate, 5-dehydro-D-gluconate, glucose-1-phosphate, deoxyadenosine, fructose-6-phosphate, or glucose-6-phosphate were supplemented to the base medium. Also, the ME-matrix improved the predictions for seven nitrogen sources, but did not correctly predict the growth phenotype for eight other nitrogen sources.

In general, false negative growth predictions indicate missing reactions in a network. No reactions were removed from the metabolic part when creating the ME-matrix. Therefore, the inability of the ME-matrix to grow, under conditions where iAF1260 could grow, was caused by stoichiometric synthesis constraints and/or constraints that couple synthesis and utilization. For instance, one of the carbon sources that did not support growth of the ME-matrix was formate, which showed weak growth *in vivo* and in *i*AF1260 [5]. We tested growth of the ME-matrix at various formate uptake rates but no growth could be observed *in silico*. It is likely that the maintenance cost of the macromolecular machinery in the ME-matrix is too high in formate minimal medium condition to support *in silico* growth. Taken together, our results show that the growth phenotype of the ME-matrix was comparable with the metabolic reconstruction of *E. coli*. As the metabolic reconstruction served as foundation for the ME-matrix, comparable growth phenotypes were expected.

#### Gene deletion analysis

**Figure 3:**
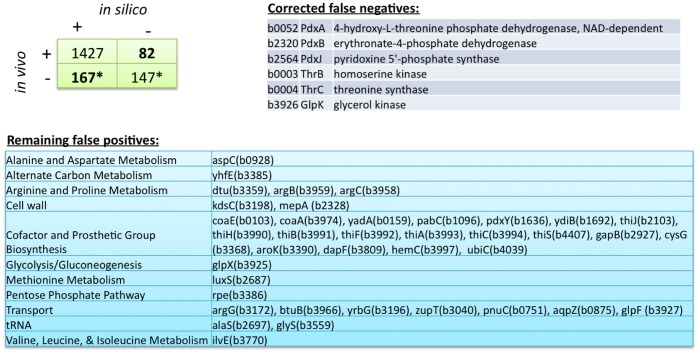
Gene essentiality. Comparison of gene essentiality in the ME-matrix, in the *in vivo* candidate essential gene list reported in [32], and the metabolic network used in Joyce et al. [64].

In order to assess the predictive potential of the ME-matrix for genome-scale gene deletion studies, we determined the *in silico* growth phenotypes for single gene knockout strains in glycerol minimal medium. Of the 1823 protein coding genes contained in the ME-matrix, 17% (314/1823) were predicted to be essential. We compared the computed gene essentiality with *in vivo* essential genes based on the Keio collection by Baba et al. [32]. In that study, the authors reported 300 candidate essential genes, 75% (229/300) of which were covered by the ME-matrix. Overall, we predict 114 essential genes and 1427 non-essential genes correctly (Figure 3). The overall accuracy of our prediction is 86%. Of the 82 false positive predictions, 16% (13/86) have been either reported as non-essential or not tested or non-conclusive in three other data sets, which the authors used for comparison of their gene list. For the true positive prediction, we could only identify three of such cases (2%).

Many of the false positive predicted essential genes involve genes, whose gene products may improve *E. coli*’s fitness under certain environmental conditions, but its absence is not crucial for growth. These gene products include, for example, the trigger factor (b0436, tig), which assists in folding the nascent peptide chain exiting the ribosome, as well as many ribosomal subunits and proteins involved in ribosomal protein, rRNA, and tRNA modification. The *in silico* essentiality can be explained by the requirement of these proteins for the corresponding reactions. For instance, we did not represent the translation termination with and without the presence of the trigger factor, as for obvious energetic reasons the model would choose to not synthesize the protein and thus the model would represent less accurately *E. coli*’s biology.

Furthermore, when we compared our predicted gene essentiality in glycerol medium with experimental data for glycerol minimal medium and the metabolic network only (*i*AF1260), our predictions agreed in 89% of the cases (Figure 3). Interestingly, the ME-matrix improved prediction of six essential metabolic genes (Figure 3), which were non-essential *in silico* when the metabolic network was used alone [5], [33].

Finally, we retrieved from the DrugBank database [34] 69 antibiotic drugs, which target 36 *E. coli* genes. The ME-matrix accounts for 33 of these drug targets, of which ten were true positive essential genes in the minimal glycerol medium analysis. The remaining 23 drug targets were predicted to be non-essential under the simulation condition, of which seven were false negatives. While the model does not predict an effect on growth capabilities for these 23 antibiotic target, it is expected that they impact other cellular functions, such as membrane integrity, which our model does not cover.

#### Reduced cost of optimal solutions in the ME-matrix

Reduced cost is a variable in linear programming (LP) problems, which nonzero for each network reaction (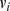) that reaches an upper or lower bound at optimality. It represents the amount by which the objective function (e.g., growth rate) could be increased when the flux rate through this reaction would be increased by a single unit [35]. In this study, we use the reduced cost to identify constraining reaction bounds in the model. We analyzed the reduced cost of the four simulated conditions for the ME-matrix (Figure 2 A). We found that the transcription initiation reactions of the rRNA operons had the greatest reduced cost associated in all four conditions. Moreover, in all simulation conditions, we placed an upper bound on the maximal possible transcription initiation rate for transcription units encoding for ribosomal RNA. The reduced cost analysis identifies these imposed constraints as limiting for achieving higher growth rates. This result highlights the competition for metabolic precursors between the ribosome synthesis and biomass production.

#### Prediction of *in silico* gene expression profile

We also wished to validate the predictive potential of the ME-matrix for the computed transcriptome. We determined the effective *in silico* codon usage by calculating the codon usage for all ME-matrix genes (Figure S3) and multiplying it by average translation rates across the 170 environmental conditions (as defined by the Biolog data, see above). The effective *in vivo* codon usage was determined similarly by multiplying the codon usage by the average expression level for each gene across various environmental and genetic conditions [36]. We found a high correlation between *in silico* and *in vivo* codon usage (Pearson correlation, 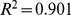) (Figure 2C). These results suggest that our predicted codon usage is physiologically relevant.

### ME-matrix Genes are Highly Conserved Across Enterobacter and Non-enterobacter Species

#### Conservation of genes in enterobacter and non-enterobacter species

**Figure 4:**
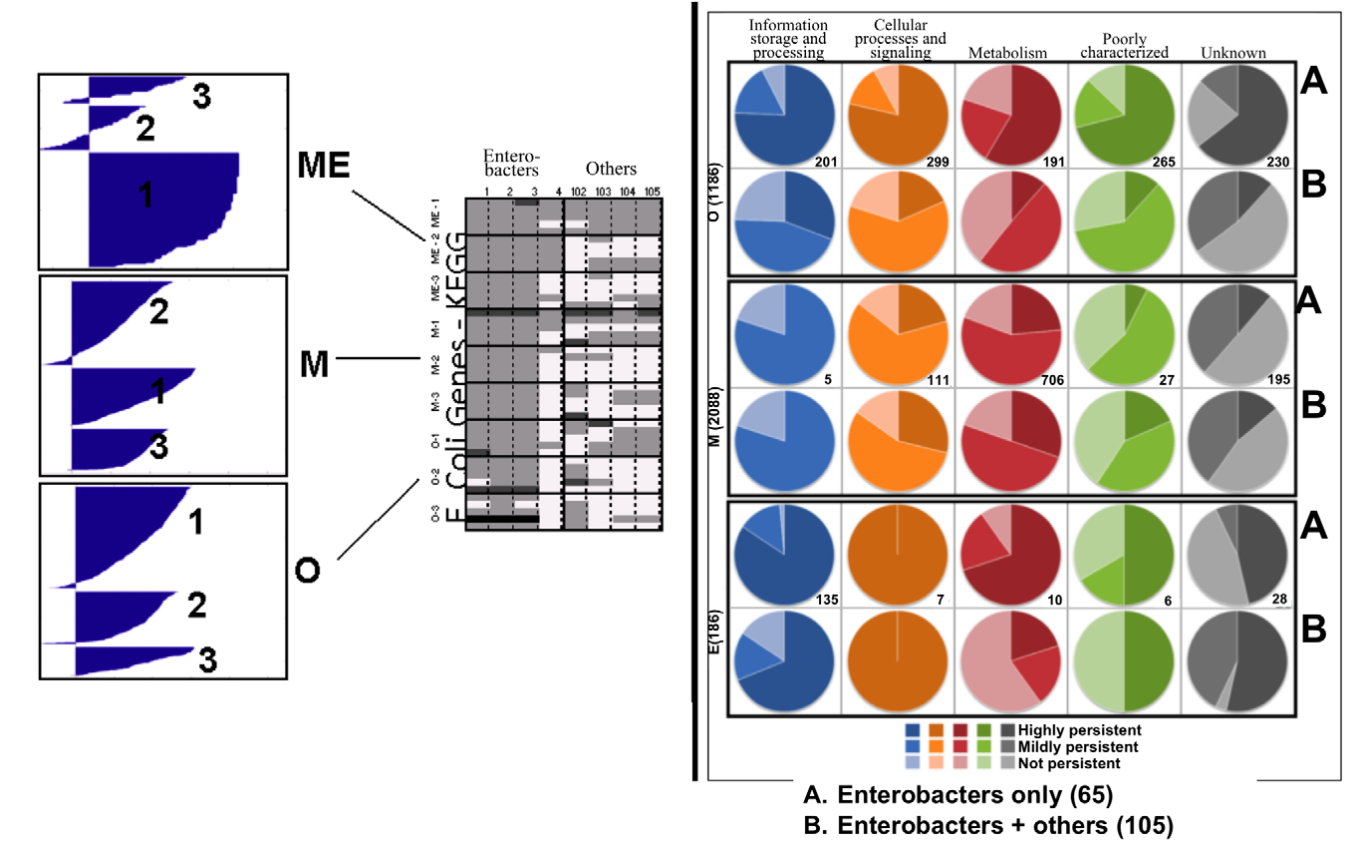
Conservation of ME-matrix genes across enterobacters and non-enterobacters. Left : K-means clustering was used to group KO terms within each category. The cluster with highest occurrence in all species were classified as highly persistent, while the next cluster was deemed moderately persistent, and finally, non-persistent. **Right**: Characterized each cluster groups by mapping to COG functions. The number of genes per gene group (O, M, E) is given in parenthesis. The number per gene with a particular COG classification is given for each gene group. (UP - highly persistent among 105 bacteria; EP - highly persistent among 65 enterobacters but not among 105 bacteria; SP - mildly persistent among 65 enterobacters and not persistent among 105 bacteria; NP - not persistent in any group of species.).

We aimed to determine genes in metabolism and macromolecular synthesis pathways that are persistent across enterobacter species as well as persistent in other, less related bacteria. We identified homologous genes to *E. coli* genes in 65 enterobacter genomes and 40 non-enterobacter bacteria by using KEGG automatic annotation system (KAAS) [37], [38], while only considering bi-directional hits. KAAS uses KEGG Ontology (KO) to define the function of a gene product. A total of 4,131 genes of *E. coli* K12 MG1665 could be assigned with 2,418 unique KO terms. Overall, the percentage of genes per organism homologous to *E. coli* ranged from 93% in *Buchnera aphidicola 5A Acrysiphonpisum* to 15.1% in organism *Staphylothermus marinus F1* NC 009033 (Table S1). Using the KAAS results, we created an orthologous gene table of the 4131 *E. coli* genes and the 105 different organisms by noting the number of orthologous genes found in a particular species (Figure 4). We categorize the homologous genes in the orthologous gene table into three groups: lifestyle genes (M) if the orthologous *E. coli* gene products were part of the metabolic reconstruction (*i*AF1260), core machinery genes (E) if the gene products were part of *E. coli*’s macromolecular synthesis machinery reconstruction, and O for all other gene products (Figure 4). We then calculated three k-means clusters using the Hamming distance (using a binary version of the orthologous gene table), which minimizes the sum of the squared distances to the centroids of these clusters. We then classified the clusters according to the mean percent occurrences of the KO groups within defined as “highly”, “mildly”, and “not” persistent. First, we considered only enterobacters in the orthologous gene table for the clustering analysis. The KO groups that are classified as core machinery coding (E) generally forms tighter clusters than KO’s, which are unclassified (O) or in the metabolic subgroup (M) (Figure S4A). However, the clusters of lifestyle coding KO groups (M) have standard deviations of 9% and 14% for “mildly” and “not” persistent groups, thus these groups are not well defined while the “highly” persistent cluster is tighter with only 3% standard deviation. When the non-enterobacters were included in the clustering analysis, the number of KO’s increased for O coding KO groups, while the E and M coding KO groups remained similar (Figure S4B).

#### Classification of persistent gene functions

We were interested to see whether there were particular cellular functions within each group (O, M, E) that had more persistent KO groups than others. We determined the COG classification for each *E. coli* gene and transferred it to the KO groups (Figure 4). In general, we found that the tendency of persistence level within the different gene groups and COG categories was conserved across enterbacters and all species. Interestingly, the genes in the O group were almost evenly distributed within the five COG classifications. While the majority of the genes in the five groups were highly persistent within the enterobacters, less than a third of these genes remain persistent within most of the analyzed species. Notably, 39 genes of the ‘poorly characterized’ and 69 genes of the “unknown” COG groups were persistent in most of the analyzed species (deemed as highly persistent). Within the KO group of lifestyle coding genes (M) there were only five KO groups of genes with a COG classification of “information storage and processing”, which were either moderately or not persistent in both cases, when enterobacter were only considered and when species across the phylogenetic tree were considered. Less than 15% of the lifestyle KO groups with a COG classification “metabolism” were highly persistent within the enterobacter highlighting the metabolic diversity of this family, while about 20% of the KO groups in this COG category could be classified as highly persistent across all species, indicating essential metabolic function and genes. These genes included those encoding for central metabolic enzymes. Knowledge of these “core” metabolic genes would allow the design of a minimal metabolic network. Interestingly, 27 of the metabolic genes included in *E. coli*’s metabolic reconstruction belong to the COG category “poorly characterized” and 195 were “unknown”, again with a large fraction of mildly persistent genes. Most of the core machinery genes (E group) belonged to the COG category “information storage and processing”, of which more than 80% were highly persistent in enterobacters and more than two thirds were highly persistent within all species. Given the fundamental functions of these genes within the cell (e.g., transcription and translation) this high degree of persistency is not surprising. Overall, this group has the largest number of persistent genes in enterobacters and in all species. Only few genes within the COG category “metabolism” and “poorly characterized” appear to be species specific.

### Predicting Constraints on Codon Usage Pattern

**Figure 5:**
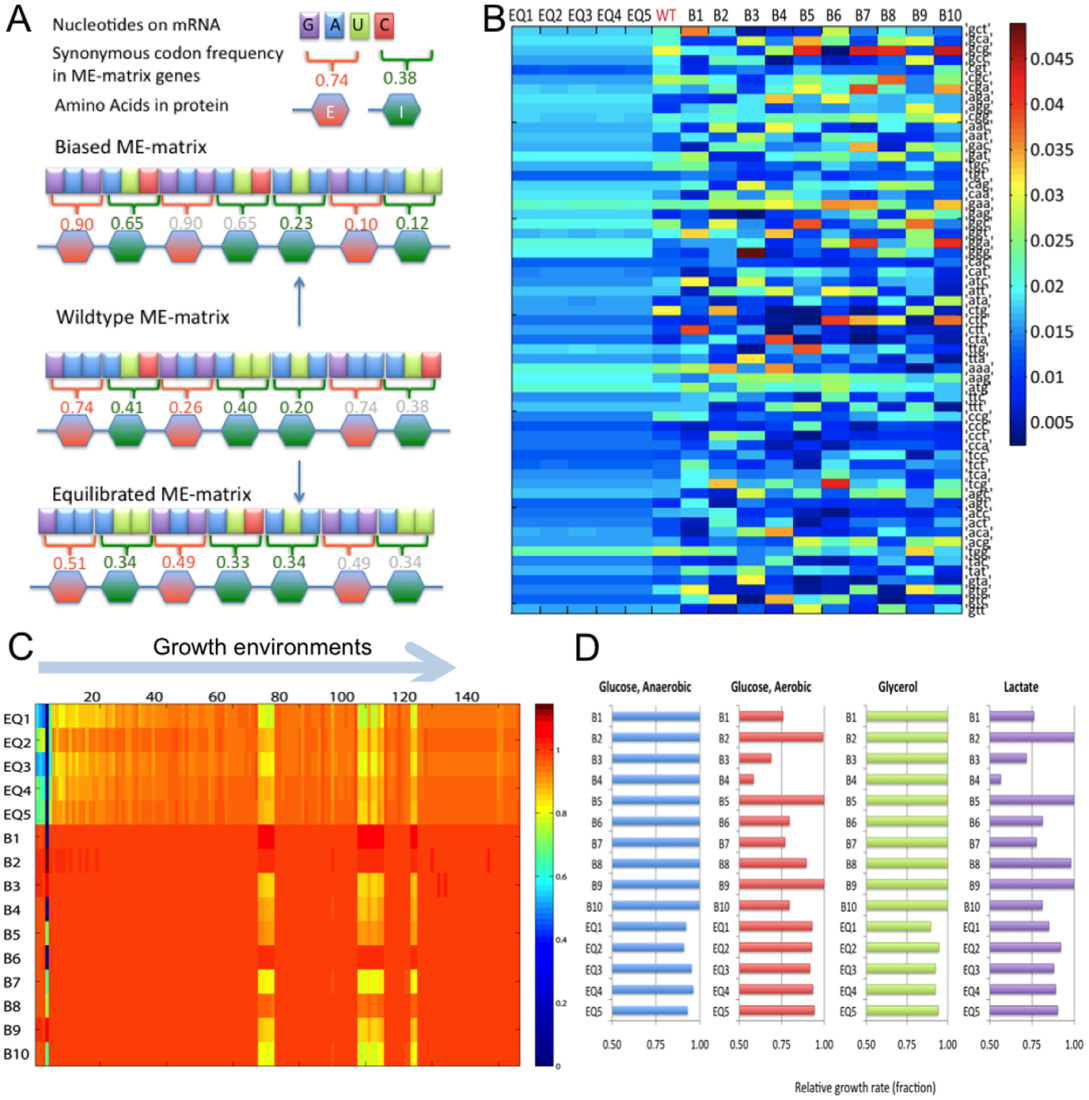
Properties of *in silico* strains. A: Differences between CUB of wildtype and perturbed ME-matrices. **B:** Heatmap of the usage of 61 codons (including start codon) in wildtype and mutant strains. **C, D:** Relative growth rates achieved by *in silico* strains across 170 environmental conditions (C) and when measured SUR and OUR were chosen as constraints (D).

**Figure 6:**
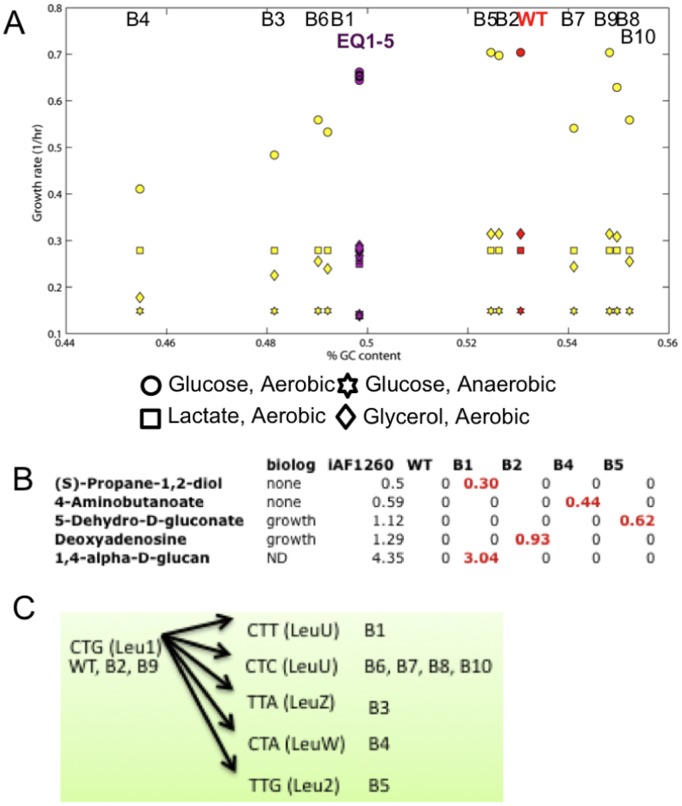
Distinguishing features between the *in silico* strains. **A:** GC content versus *in silico* growth rates. **B:** Growth rates in conditions, where the wildtype could not grow but biased strains and the metabolic model, *i*AF1260 [5], did. **C**: Changes of major leucine codon in the CUB perturbed ME-matrix. tRNA recognizing the codon is given in parenthesis. Leu1 and Leu2 are generic tRNA species representing multiple leucyl-tRNA species, see Table S2, and [23].

A key interest of systems biology is to develop a mechanistic basis for the genotype-phenotype relationship. The ME-matrix explicitly captured the nucleotide sequence for almost 2000 genes and stoichiometrically represented their cellular functions, so we addressed the question if and how the codon usage bias (CUB) may evolve to maximize growth rate in different growth environments. We generated a range of perturbed ME-matrices differing only in their codon usage from the wildtype ME-matrix; ten ME-matrices with more biased codon usage (“biased strains”, B1–B10) and five ME-matrices with less biased codon usage (“equilibrated strains”, EQ1-EQ5) (Figure 5A). As expected, the CUB of the equilibrated strains was highly correlated while the CUB was idiosyncratic in the biased strains (Figure 5B). With FBA, we calculated the strains’ growth rates across the aforementioned 170 conditions. The growth rates of the biased strain models were comparable to the wildtype model, while the equilibrated strains grew slower (Figure 5C). We identified five cases, where a biased strain was able to grow, but the wildtype was unable to grow (Figure 6B). Thus, these strains exhibited an increased fitness in these conditions. We also calculated growth rates using experimentally measured carbon and oxygen uptake rates as boundary conditions [24], [31] (Figure 2A). Seven biased strains exhibited up to 50% reduction in growth rates in two conditions, while the equilibrated strains showed reduced growth rates in all four conditions (Figure 5B). These results demonstrate that, *in silico*, changes in CUB can alter a strain’s ability to grow in certain environments and affect the growth rate. How is this possible given that perturbation to codon usage only affects stoichiometric coefficients and not the sparsity pattern of the perturbed ME-matrices as no reactions were added or removed?

#### GC content analysis of the *in silico* strains

Statistical genomic studies have identified GC content as the single most informative determinant of CUB [39], [40]. A recent study showed that CUB, but not GC content, correlated with minimum generation time [41]. We examined the relationship between GC content, CUB and growth rate in wildtype and perturbed ME-matrices. Generally, we found that GC content correlated with growth rate but there was a plateau at a wildtype growth rate (Figure 6A). At higher GC content, CUB seemed to dominate the calculated maximal possible growth rate. The GC content of perturbed strains was between 45% and 55%, which is similar to wildtype (53%). Therefore, the metabolic changes due to GC content changes were minor.

#### Shannon entropy analysis of codon usage in the *in silico* strains

We computed the Shannon entropy of the codon usage for each *in silico* sequence reflect how biased or unbiased the codon usage of the 1,823 genes in the strains is compared to a random distribution (Figure S5). As expected, the equilibrated strains had the highest entropy (the most random sequence with least codon bias), while the biased strains had lower entropy than the wildtype strain (Figure S5). No obvious correlation between entropy value and maximal achievable growth rate could be observed, except that high entropy seemed to reduce the growth rates in some conditions (Figure S5).

#### Reduced cost analysis of optimal growth states of the *in silico* strains

The ME-matrix allows one to derive causal hypotheses by analyzing numerical properties of FBA solutions, such as the reduced cost. Each mutant ME-matrix had bounds identical to the wildtype: i) upper bounds on each transcription (initiation) reaction, i.e., RNA polymerase elongation rate times the gene dosage, ii) upper bounds on the sum of tRNA synthesis rates, iii) an upper bound on uptake rates of carbon source and/or oxygen, and iv) a lower bound on non-growth associated ATP maintenance. When analyzing the reduced cost vector associated with each optimal solution, we found that the growth of biased strains in glycerol and glucose (anaerobic) growth conditions was limited by upper bounds on ribosomal RNA (rRNA) operon transcription reactions, as was the case for the wildtype (see above). In contrast, for all seven biased strains in lactate and glucose (aerobic) conditions, the bounds on leucyl-tRNA transcription reactions were limiting growth (Figure 6C, Figure S6). Leucine is the most abundant amino acid in *E. coli*’s genome, which encodes for eight leucyl-tRNAs, five of which can read the most frequent codon CTT (Table S2). In all biased strains with reduced growth rate, the change in CUB shifted CTT to minor ones, which are read by single tRNA species (Figure 6C). Five biased strains had a highest reduced cost for the 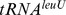 transcription reaction. In the case of strain B2, the growth limiting tRNA species was condition dependent. We numerically confirmed that relaxation of bounds corresponding to the highest reduced cost was sufficient to restore wildtype growth rate and therefore that rRNA transcription limited growth. The growth rates of equilibrated strains were limited to a similar degree by bounds on many different tRNA transcription reactions. These results show that tRNA supply was growth limiting in the perturbed ME-matrices.

## Discussion

In this study, we created the first sequence-specific, integrated model of metabolism and macromolecular synthesis for the model organism *E. coli*. Using different computational tools, we assessed the predictive potential of the ME-matrix by comparing model prediction with published experimental data. In general, we found similar or improved predictive potential when compared to the metabolic network alone. We then employed the ME-matrix to assess the impact of CUB on the growth phenotype under different environmental conditions and found that the tRNA availability was growth limiting in most growth conditions for the biased strains but not for the wildtype or equilibrated strains.

When comparing the quantitative predictive potential of the ME-matrix with experimental growth rates in four well defined environmental conditions, we found that the ME-matrix model outperformed *i*AF1260 (Figure 2A). We also obtained good agreement with the Biolog experimental data reporting growth capability in 170 defined minimal media (Figure 2B). These results gave us confidence in the ME-matrix’ growth phenotype predictive capacity in a broad range of conditions. Similarly, the ME-matrix model predicted correctly the knockout growth phenotype in the majority of the cases. Interestingly, the ME-matrix predicted correctly the essentiality of six genes, which were non-essential in *i*AF1260 (Figure 3) [5], [33]. This example underlines that the functional coverage of *i*AF1260 is too limited to account for all observed growth phenotypes in different environmental and genetic perturbations. Thus, expanding current models to whole cell models will further increase the model’s accuracy besides augmenting its functional coverage [42].

Stoichiometric coefficients in the ME-matrix are distributed over four orders of magnitude as many metabolic precursors were required to form one macromolecule (Figure 1B). For example, the transcription of an average gene requires 1000 nucleotides to produce one mRNA molecule. Metabolism provides precursors for macromolecular synthesis, which in turn synthesize the metabolic enzymes that catalyze biochemical reactions (Figure 1A). We linearly approximated this interdependency with linear inequalities that couple steady state reaction rates distributed over many orders of magnitude (i.e., 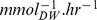 vs. 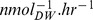). When conducting flux balance analysis, the combination of stoichiometric coefficients and steady state reaction rates distributed over many orders of magnitude give rise to an ill-scaled linear optimization problem. Naive application of off-the-shelf linear optimization software to solve for a steady state in a multiscale model can causes software to erroneously report infeasibility or return an inaccurate flux vector violating standard tolerances set to ensure numerical accuracy. To overcome this issue, we developed a new technique to pre-process a multiscale flux balance analysis problem and tune solver parameters such that accurate and optimal steady states can be computed. For further details and links to open source code, see [43].

When we compared the ME-matrix gene conservation across enterobacter and non-enterobacter species, we observed that many are highly conserved, as one would expect due to their central function in key cellular processes (Figure 4). *E. coli*’s metabolic reconstruction has been successfully used as a baseline for reconstructing four other *E. coli* strains by adding maximally eight reactions to the *E. coli* reconstruction, while up to 66 reaction were removed [44]. Similarly, metabolic reconstructions have been assembled for closely related organisms starting from the *E. coli* reconstruction, such as *Salmonella typhimurium* LT2 [8], [45] and *Klebsiella pneumonia* [46]. Lifestyle signatures are characterized by a set of persistent but non-essential genes. The paleome is said to form three sets of clustering genes: 1) core machinery genes, 2) genes permitting cell division, and 3) genes, which are poorly clustered but code for the basic building blocks of cells [47]. It has been suggested that the minimal genome set can be calculated from the paleome. Thus, highly persistent *E. coli* genes could be used to determine the minimal number of reactions necessary for modeling a large number of other related or even less related organisms. Lifestyle coding genes and reactions could be added later to model specific behaviors of the target species. The minimal genes set could be an extremely useful tool in synthetic biology for creating new model organisms and even predicting the pathways of evolution [48]. Moreover, the high conservation of the ME-matrix genes is particularly interesting as the ME-matrix accounts for all major antibiotic targets, except DNA gyrase [49], which could be exploited for functionally assessing lethal or sub-lethal antibiotic doses and combination therapies of novel antibiotic substances using the ME-matrix of *E. coli* or other phylogentically related organisms.

After assessing the predictive potential of the model and the conservation of its genes, we employed the ME-matrix for the analysis of constraints on the codon usage in various environmental conditions We found that the wildtype’s rRNA, but not tRNA, transcription was growth limiting in the tested environments. This result is in agreement with experimental data reporting correlation between ribosome number and growth rate [50]. The wildtype CUB and tRNA supply must therefore be complimentary to the tRNA demand for each of the tested environments. Reduced or no growth of a perturbed ME-matrix in an environment was caused by an imbalance of this demand-and-supply relationship for some tRNA species, as not all proteins required to sustain the growth could be synthesized (Figure 6C). The identification of growth enabling perturbed ME-matrices (Figure 5B) suggests that the wildtype operon structure was consistent with CUB of co-expressed genes in most but not all environments. Our results indicate that CUB reflects environments that an organism can occupy, which agrees with statistical genomic studies [41], [51]. Upon CUB perturbation, an increased tRNA demand may be met by augmenting supply that could be achieved by i) genome re-organization to relocate tRNA genes closer to the origin of replication, which would increase the gene copy number via gene dosage effect; ii) acquisition of tRNA genes from other organisms; or iii) modification of a tRNA to expand its set of read codons. It has been recently shown that a second leucyl-tRNA (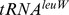) is able to read CTT in *E. coli* MAS39 due to a uridine-5-oxyacetic acid modification [52].

It remains to be established that 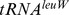 in *E. coli* MG1655 can also read CTT. This example demonstrates that the ME-matrix reconstruction has the potential to elucidate lack of robustness, and thus may assist in the generation of novel hypothesis and subsequent experimental studies.

Two non-exclusive hypotheses have been proposed to explain co-evolution of CUB and tRNA content [53]–[55]: i) the mutational (neutral) hypothesis proposes that mutational processes without any associated loss or gain of function occur (e.g., through changes in cellular nucleotide content [56] leading to changes in CUB); and ii) the natural selection hypothesis suggests that synonymous mutations affect the fitness of the organism and manifest in CUB across the genome or genes [53], [57]. Current empirical and experimental evidence provides support for both hypotheses, also known as mutation-selection-drift balance theory of CUB [55]. To date, no comprehensive conceptual framework exists to investigate the link between CUB and tRNA content and its effect on protein synthesis, growth phenotype, and possible growth environments
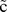
iteVieira:2010. The CUB perturbations in the *in silico* strains affected all ME-matrix genes equally and thus the strains satisfy the mutational hypothesis. Our predictions demonstrated that due to tRNA supply shortage, the metabolic requirements for a proteome sufficient to sustain growth was not attainable. The adjustment of this shortage through expansion of tRNA content or reading is most consistent with the natural selection hypothesis. We identified reduced and increased maximal growth rates of the *in silico* strains depending on environmental niche consistent with previous observations that synonymous codon usage significantly impacts achievable growth phenotypes [58]. Using a genome-scale analysis framework that is novel to molecular systems biology, we provide an explanation of how expansion of tRNA content and/or reading may be used as an evolutionary mechanism to deal with mismatches between CUB (genotype) and environment to maximize growth rate (phenotype).

Network reconstruction technologies developed over the past 20 years enabled us to build an integrated metabolic, macromolecular synthesis reconstruction for *E. coli* K12 MG1655. This ME-matrix is a knowledge-base and it can also be used for computations enabling the simultaneous reconciliation of the activities of its gene products. The models derived from the ME-matrix reconstruction will enable a new dimension of biotechnological, biomedical, and evolutionary applications that could not been addressed with conventional modeling approaches. Other applications may include protein engineering and prediction of cellular proteome. This ME-matrix formalism represents a milestone towards cell-scale modeling to achieve this ambitious goal in the near future.

## Materials and Methods

### Constraint-based Reconstruction and Modeling Approach

A reconstructed biochemical network is often represented in a tabular format, listing all network reactions and metabolites in a human-readable manner (see [3] for details). The conversion into a mathematical, or computer-readable format, can be done automatically by parsing the stoichiometric coefficients from the network reaction list (e.g., using the COBRA toolbox [59]). The mathematical format of the reconstruction is called a stoichiometric matrix, or 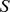 matrix, in which the rows correspond to the network metabolites and the columns represent the network reactions. For each reaction, the stoichiometric coefficients of the substrates are listed with a minus sign in the corresponding cell of the matrix, while the product coefficients are positive numbers. The resulting size of the 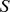 matrix is 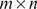, where 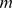 is the number of metabolites and 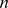 the number of network reactions. Mathematically, the 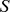 matrix linearly transforms the flux vector



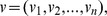
.to a vector of time derivatives of the concentration vector



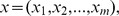
as



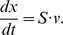



At steady-state, the change in concentration as a function of time is zero; hence, it follows:
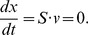



The set of possible flux vectors 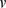 that satisfy this equality constraint might be subject to further constraints by defining
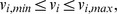
for reactions 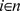. In fact, for every irreversible network reaction 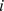, the lower bound was defined as 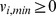 and the upper bound was defined as 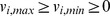. Exchange reactions supply the network with nutrients or remove secretion products from the medium. The uptake of a substrate by the network was defined by a flux rate 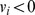 and secretion of a by-product was defined to be 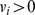 for every exchange reaction 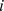. Finally, the application of constraints corresponding to different environmental conditions (e.g., minimal growth medium) or different genetic background (e.g., enzyme-deficient mutant) allow the transition from biochemical network reconstruction to a condition-specific model. Note that the network reconstruction is unique to a target organism (and defined by its genome) while it can give rise to many different models by applying condition-specific constraints. In this study, all flux rates are given in 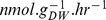 if not stated differently.

#### The metabolic reconstruction of *E. coli*

The metabolic reconstruction of *E. coli*, *i*AF1260 [5], was obtained in SBML format (Ec_*i*AF1260_flux1.xml), from http://systemsbiology.ucsd.edu) and imported into MATLAB (MathWorks, Inc.) using the COBRA Toolbox [59]. *i*AF1260 accounts for 1,260 *E. coli* genes and 2,077 reactions, including 1,339 unique metabolic reactions, 690 transport reactions, and 304 exchange reactions [5]. 1,294 reactions have gene-protein-reaction associations. *i*AF1260 accounts for 1,039 unique metabolites. A total of 1,148 unique, functional proteins are accounted for including 167 multigene complexes and 346 isozymes [5]. Prior to merging *i*AF1260 with the ‘E-matrix’, all gene associations connected to the artificial gene ‘s0001’ were removed. *i*AF1260 contains tRNA charging reactions that were also removed from the model before integration.

#### The macromolecular synthesis machinery of *E. coli*

The macromolecular machinery reconstruction, deemed expression or ‘E-matrix’, was downloaded and imported into MATLAB [23]. It accounts for 249 transcription units containing 423 genes, 228 proteins (34 without coding gene), 86 tRNA species, 22 rRNA species, and one miscellaneous RNA species. A total of 11,991 network components and 13,694 reactions describe the synthesis, assembly, and function of the macromolecular synthesis machinery of *E. coli* K12 MG1655. The bounds on exchange and transport reactions for metabolites, which were present in the E-matrix and in *i*AF1260, were set to be zero (lower and upper bound) in the E-matrix prior to integration.

#### Construction of transcription and translation reactions for metabolic enzymes

The integration of the E-matrix with *i*AF1260 requires that all metabolic enzymes (1260 gene products) are synthesized by the network. Therefore, we created template reactions for transcription, translation, mRNA degradation, etc. as well as the gene information (e.g., transcription unit assignment from EcoCyc [60], gene coordinates, and gene direction from [61]) (see Table 1 for a complete list). The formulation of the reactions for individual genes and transcription units was done in an automated fashion as described elsewhere [23].

#### Reformulation of M-matrix reactions

Consider the following sample reaction G6PP from the M-matrix:

– 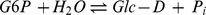



This equation can be changed by adding enzymatic complexes. First, information was collected about the reaction (G6PP):

– Gene loci = b0822, gene = ybi, and protein = YbiV

Second, the reaction was converted into the following one (notice the name change):

– 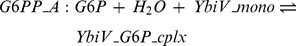



Third, new reactions were added to the reaction list.

– 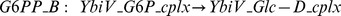



– 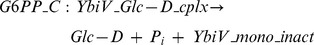



– 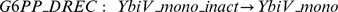



If a reaction was reversible (which G6PP is), the reactions for the reverse direction were as follows:

– 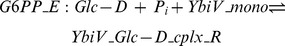



– 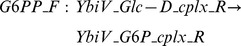



– 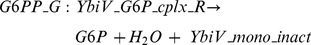



If the equation occurred in the periplasm [p] or extracellular space [e], a corresponding transport reaction was also included. The ME-matrix does not capture protein secretion mechanisms. The reaction G6PP is located in the cytoplasm, not requiring transport reactions, however, hypothetical transport reaction(s) would be as follows:

If in the periplasm:

– 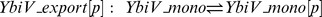



If in the extracellular space:

– 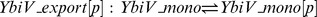



– 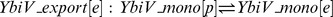



The sample reaction represented a one gene to one protein relationship. There are three further possible gene-protein-relationships.

- *The “OR” case.* Two or more different genes could code for proteins, which could catalyze the same reaction(s) (isozymes). In this case, each gene was assigned to the reaction as shown above. Therefore, if the G6PP reaction could be catalyzed by 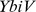 and some other protein (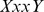), we would create the reactions listed above and also repeat the process with 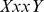. In this case, the naming convention for reactions was also adapted to reflect the protein catalyzing the reaction. Thus, instead of using 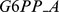, 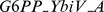 and 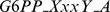 would be used.
- *The “AND” case.* Multiple genes could code multiple proteins that must form a complex to catalyze the reaction. In this case, an additional reaction was created representing the complex formation reaction. Suppose 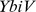 and 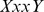 are both required for 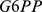. A corresponding complex formation reaction would be created:

– 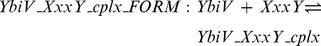



This new complex would then be used in the reactions above replacing 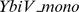.

- The third instance was the combination of both the “OR” and “AND” case. The rules laid out above were used to combine the two.

The integration of reformulated metabolic reconstruction and the extended E-matrix was done computationally by creating a non-redundant reaction list containing the union of the two reconstructions.

#### Protein complex formation

Information about protein complex formation was obtained from *i*AF1260, which describes the relationship between gene products and metabolic reactions in terms of Boolean logic [5]. This information was complemented with protein complex information obtained from EcoCyc [60] and primary literature. Protein complex formation reactions for multimeric proteins were formulated manually assuming that all subunits bind simultaneously in a composite reaction. A monomeric subunit was assumed when no information was available.

#### Metallo-ions and prosthetic groups

Information about metallo-ion and/or prosthetic groups were obtained from EcoCyc [60], protein structures of *E. coli* enzymes and primary literature. The information was manually assembled, while the network reactions were formulated based on the template reactions (see [23] for details). If no information about the number of associated ions could be found, we assumed one ion per monomer.

#### Adjustment of biomass

The amino acid and growth associated maintenance (GAM) of the *E. coli* biomass reaction in the ME-matrix was adjusted to account for the cost of synthesis of the machinery and proteins in the ME-matrix. After performing a sensitivity analysis for these two parameters, we adjusted the biomass reaction to account for 50% of the amino acid content and 50% of the GAM of the biomass reaction in the metabolic reconstruction. The adjusted biomass reaction was used in all simulations if not noted differently.

#### Coupling constraints

There were three dominant sets of constraints applied to the ME-matrix.

1. Constraints on the exchange reactions to simulate different environmental conditions.
2. Constraints on the maximal transcription rate for stable and messenger RNA - these two set of constraints are on 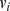: 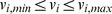

3. Coupling constraints on reactions, in the form of
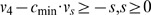
(1)

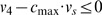
(2)

where 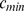 and 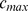 are the bounds on the proportion factor or ‘coupling coefficient’. 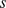 can be used to allow accumulation of a network component without using it in the steady-state solution thereby relaxing the requirement that all synthesized network components needed to be used within the network. Eq. 2 ensured that a higher flux through 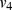 raises the lower bound on the synthesis reaction 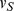. These linear inequality coupling constraints retained the numerically scalable character of FBA.

Coupling constraints were added:

- between mRNA synthesis and translation (via _mRNA_degr1 and _CONV2):
  – The parameters 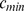 and 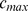 are determined based on the following equation
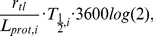
(3)where 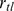 is the translation rate at a given doubling time (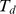), 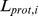 is the length of the protein i (in amino acids), and 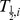 is the half-life time of mRNA i. The upper bound on half-life time was assumed to be 60 minutes, while the lower bound was set to be 0.1 minute.
- between protein synthesis and protein utilizing reactions

  – for E-matrix proteins: via DM_ and _RECYCL

* The parameters 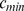 and 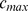 were set to 1 and 10000, respectively. This parameter corresponds to the turnover/utilization rate of the protein. Note, that the proteins were not degraded in the network, as they were considered to be stable, which is a valid assumption in respect to the doubling times considered here.

  – for M-matrix proteins (metabolic enzymes): via DM_ and _DREC

* The rational behind the parameters 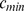 and 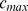 was similar to the E-matrix proteins.

* In general, we observed that the 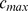 should be chosen higher for enzyme as the metabolic flux rates were much larger than the synthesis reaction rates, and thus either more protein (thus higher protein synthesis flux) should be necessary or a higher activity (utilization rate) should be required to meet this increased demand.

- between tRNA charging and utilization (see [28] for details).

Note that the demand reactions represent the “real” accumulation of proteins observed in cells, which may be measured by proteomic approaches. Hence, proteomic data could be directly mapped onto demand reactions. The situation is quite different for the transcripts where the turnover was modeled but no “real” mRNA accumulation was permitted. A temporary accumulation was modeled using artificial reactions (‘_CONV2’), which allowed each mRNA species to cycle within the network for a limited time before getting turned over. Hence, the mRNA synthesis corresponds to the maintenance of the internal pool for each transcript within the cell. In fact, Eq. 3 controled the pool size for each transcript within the network. There is a direct conversion possible from _CONV2 and _mRNA_degr1 allowing to back-calculate half-life time and mRNA concentration for each transcript given a flux vector [28].

#### Simulation constraints

Experimental measurements of substrate and oxygen uptake rates were applied on the exchange reactions (Figure 2A). The unit of the ME-matrix is 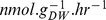. The maximal reaction rates of stable RNA synthesis were constrained as described in [23]. The maximal reaction rates of mRNA synthesis were constrained using the same approach but changing the mRNA transcription elongation rate [50]. In all simulations, the non-growth associated maintenance (ATPM) requirement was set to 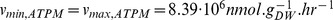 as defined in [5]. The ribosome production rate (DM_rib_50) and the biomass reaction (Ec_biomass _*i*AF1260 _core_59p81M) were unbounded. The base medium allowed the free uptake of the following compounds by setting their corresponding lower bound to 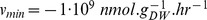 **:**

EX_h2s(e), EX_ca2(e), EX_cl(e),EX_co2(e), EX_cobalt2(e), EX_cu2(e), EX_fe2(e), EX_fe3 (e), EX_h2o(e), EX_h(e), EX_k(e), EX_mg2(e), EX_mn2(e), EX_mobd(e), EX_na1(e), EX _tungs(e), EX_zn2(e), EX_cbl1(e).

#### Growth comparison with Biolog and *i*AF1260

Biolog data for *E. coli* K12 MG1655 were downloaded from the company's website (http://biolog.com). A total of 170 tested compounds were in the reconstruction. The oxygen consumption rate was set to 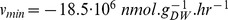 and 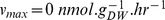. Each nutrient was added to the base medium by setting the corresponding uptake rate to 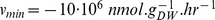 in the case of carbon sources, and 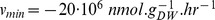 in the case of nitrogen, phosphorus, and sulphur sources. Default elemental sources were as follows: D-glucose as carbon source, ammonium ion (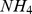) as nitrogen source, orthophosphate (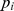) as phosphorus source, and 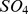 as sulfur source. The sources were added to the base medium, when the corresponding source was not tested for. Furthermore, the maximal possible transcription rates for each stable RNA transcription unit and for each protein coding gene were limited assuming a doubling time of 24 minutes, which provides an upper bound, since no information were available concerning growth rates for the different growth conditions tested in the Biolog setup. The growth results for *i*AF1260 were obtained from [5].

#### Single gene deletion study

Performing a single gene deletion study in the ME-matrix is different to the single deletion study in metabolic networks, because (i) proteins are explicit part of the metabolic reactions and (ii) transcription may occur with other genes (if co-expressed in a transcription unit), and thus coupling constraints would cause all genes in the transcription unit to not be expressed. Therefore, all translation initiation reactions for the gene were identified (e.g., ‘tl_ini_bxxx’) and the corresponding lower and upper bounds were set to zero. Then, all coupling constraints were identified and removed. We then maximized for the biomass reaction in the *in silico* knockout strain. The same procedure was repeated for all 1,823 ME-matrix genes. We compared the *in silico* growth phenotype of the single gene deficient strains for the 1,260 metabolic genes in aerobic glycerol minimal medium and with the published experimental study [33] and with the *in silico* single knockouts of *i*AF1260 (results were taken from [5]).

#### Organisms considered in for gene conservation

A total of 105 bacteria were considered in this study (see Table S1 for a complete list). 65 species were from the Enterobacteriaceae family, while further 40 bacteria were chosen from the phylogentic tree. Complete genomic protein sequences for *E. coli* and each of the bacterial species used for this study was downloaded from the NCBI database (ftp://ftp.ncbi.nih.gov/genbank/genomes/Bacteria, accessed Feb 2009.).

#### Mapping of *E. coli* genes to other bacteria

The protein sequences of the study organisms were uploaded to the KEGG Automatic Annotation Server (KAAS, http://www.genome.jp.kegg/kaas/, version 1.5a.) The KAAS service provides cross-species gene annotation based on KEGG pathways and BRITE hierarchies [37]. Briefly, during the KAAS procedure, a given list of protein sequence is queried by BLAST to the reference sequence from the KEGG database. For this study, the chosen reference set was “eco” for *E. coli*. Then, the homologs with a bi-directional hit rate of higher than 0.95 were selected and divided to KEGG Ortholog (KO) groups [37]. A KO number is then assigned to a gene based on a score obtained from calculating probability and heuristics on the homologs [37]. The output for each search was a list of query genes with the KO number given by KAAS. First, a list of 2,418 unique KO matches for *E. coli* was obtained by uploading the protein sequence files for *E. coli* to KAAS. This list provided a mapping from one or more *E. coli* gene “b” numbers (Blattner numbers) to a KO term, which allowed us to divide the 2,418 KO terms into three major subgroups: M-metabolic subgroup based on genes included in the metabolic reconstruction of *E. coli* [5], E-core machinery subgroup based on the genes included in the reconstruction of macromolecular synthesis machinery of *E. coli* [23], and O - others. An orthologous gene table was formed from counting the number of *E. coli* orthologous gene groups (KO) found in species of the Enterobacteriaceae family. The table was extended to include an additional 40 non-enterobacters and clustering was repeated on this table.

#### Clustering of orthologous genes

As a next step, we transformed the orthologous gene table into a binary table where each 1 represents the presence of an orthologous group, KO, and 0 represents no KO for that species and *E. coli*. We clustered presence/absence of orthologous gene groups within each subgroup (E, M, O) using the k-means clustering function and Hamming distance in MATLAB (MathWorks, Inc). The corresponding average silhouette widths were for E = 0.6623, M = 0.3879, O = 0.2686. Instead of delineating between persistent genes and non-persistent genes, we systematically defined persistence as three levels classified as high, mild, or not persistent.

#### Determination of persistent genes

With the addition of non-enterobacter species, genes in the KO group list were sorted based on the following definitions: universally persistent (if more than 69% of all species shares an ortholog for the E subgroup, 53% for M, and 49% for O), enterobacter persistent (if more than 78% of the enterobacter species shares this gene group, but not the non-enterobacters, for the E subgroup, 86% for M, and 49% for O), species persistent (if 76% of the species in enterobacter share the gene group but not highly persistent among enterobacter or all species level could be observed, for the E subgroup, 35% for M, and 21% for O), and non-persistent.

#### Creation of *in silico* strain library

A total of 15 *in silico* CUB mutant strains were generated consisting of ten biased strains and five equilibrated strains. The genetic code as well as the modeled tRNAs used for the formulation of the synthesis reactions in the ME-matrix are listed in Figure S1 and Tables S2. In the ME-matrix, a tRNA species could read multiple codons. Similarly, a codon could be recognized by multiple tRNA species. Generic tRNA species were added to the ME-matrix to permit overlapping recognition of tRNA species (see also [23] for details). This formulation permitted to model the complexity of tRNA reading while not requiring to write all possible alternate translation reactions. The use of generic tRNA species also highlights the redundancy in the codon reading. The CUB was perturbed as illustrated in Figure 4A by replacing a codon by one of the possible synonymous codons either i) resulting in biased strains, or ii) such that every codon has equal usage resulting in equilibrated strains.

*The biased strains were generated using the following algorithm:*

**Input:** model, sequence for each gene in model, number of iterations m

**Output:** model_biased

**Algorithm:**

1. Choose randomly a codon, 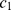

2. Identify possible synonymous codons: 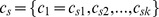

3. Choose randomly one codon from 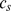: 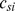

4. Replace all instances of 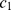 with 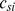

5. Update ME-matrix for all genes based on new gene sequence:
  - Transcription reactions.
  - mRNA degradation reactions.
  - Translation reactions (tRNA molecule will be updated based on codon recognition).
6. Repeat 1 through 5 m times, m = 100.

*The equilibrated strains were produced as follows:*

**Input:** model, sequence for each gene in model, number of iterations m

**Output:** model_eq

**Algorithm:**

1. Initialize vector codon = zeros, which will count the occurrences of different codons in the genome
2. Define a random order of genes to start step 3
3. For each gene 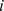 of the model genes
  - For each codon 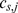 in gene sequence 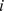

  - Identify possible synonymous codons: 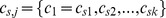

  - Choose codon 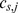 from 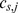 with lowest usage in vector codon
  - iv. Replace 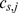 with 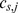 in gene sequence 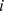

  - Update codon
4. Update ME-matrix for all genes based on new gene sequence:
  - Transcription reactions
  - mRNA degradation reactions
  - Translation reactions (tRNA molecule will be updated based on codon recognition)
5. Repeat 1 and through 4 m times, m = 100

Note that each strain had its own ME-matrix, which contained the alterations in the 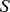 matrix but had the same reaction and coupling bounds as the wildtype. The change in codon usage was introduced to the corresponding ME-matrix by (i) adapting the nucleotide triphosphate requirements in the corresponding transcription reactions, (ii) changing the nucleotide monophosphates released in the mRNA degradation reactions, and (iii) updating the tRNA species according to the new codons (Table S2). Note that neither the start codon nor the stop codons were modified in the strains. For each perturbed ME-matrix and the wildtype ME-matrix, we applied the same simulation constraints and compared the maximal computed growth rate.

#### GC content

The GC content of the individual strains was calculated by counting the instances of guanine and cytosine residues in the 1,823 protein coding genes included in the ME-matrix. The genome sequence used for the wildtype was version m56, [62], while the modified gene sequence was used in the case of the *in silico* strains.

#### Shannon entropy

In order to quantify the degree of synonymous codon bias in a sequence, we computed the synonymous codon entropy [63]. We used the Shannon entropy function since it reaches a maximum when all codons have equal probability of coding for their respective amino acids. Conversely, the entropy reaches its minimum when each amino acid is exclusively coded for by one of its possible codons. The synonymous codon entropy, 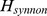, was defined as
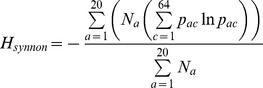
where 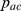 is the probability that amino acid 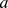 is encoded by codon 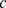, and 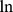 denotes the natural logarithm. If no amino acid is not coded for by a particular codon, 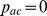, we use the definition 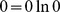. Here we weight the contribution to the total synonymous codon entropy by the number of each particular amino acid, 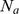, within a sequence. This means that a rare amino acid with highly biased synonymous codon usage does not overly effect the total entropy of a sequence if the remainder of the common amino acids have relatively unbiased codon usage. Since we wish to compare the synonymous codon bias between genes, we normalize the total by the total number of amino acids in a sequence, 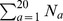. If we wish to calculate the total entropy for a set of genes then we simply sum up the synonymous codon entropy for each gene's sequence, then divide by the total number of genes. Therefore, the total synonymous codon entropy is comparable between different sequences, such as mutant biased, wild type, and mutant equilibrated strains, which have low, medium and high total synonymous entropy, respectively, (Figure S5).

#### Numerical tests

Calculating with the ME-matrix was time-consuming and numerically challenging due to the multiscale nature of the FBA problem (Figure 1B). Therefore, it was required to test each computed point if it lay within the solution space, e.g., to test if 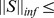 tolerance, where tolerance was 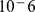, and similarly for coupling constraints and bounds on reaction rates.

All simulations were carried out in MATLAB (MathWorks, Inc.) using Tomlab (Tomlab, Inc.) as numerical analysis interface for linear programming.

The ME-matrix reconstruction used in this study is described in the Supplemental Tables and is available in MATLAB format under http://notendur.hi.is/ithiele/downloads.html.

## Supporting Information

Figure S1 **Degeneracy of the genetic code.** A. Genetic code employed in this study. Number of cognate tRNAs per amino acid is given in parenthesis. B. Schematic illustration of the degeneracy of genetic code.
(EPS)

Figure S2 **Sensitivity analysis.** We tested the sensitivity of the predicted growth rate as a function of the remaining amino acid (AA) requirement in the biomass function and as a function of the remaining growth associated maintenance (GAM) that is left in the biomass function. The experimentally observed growth rate is shown with the dotted line. Since the ME-matrix covers about 1,900 of 4,400 *E. coli* genes, we decided to allocate 50 of the AA requirements and the 50 of the GAM for the ME-matrix genes and gene products. This plot also highlights that finetuning of these two parameters will be important to obtain accurate predictions in growth rate.
(EPS)

Figure S3 **Codon usage.** Comparison of codon usage in ME-matrix associated genes and across the genome.
(EPS)

Figure S4 **Summary statistics of clustering results.** O - Unclassified, M -lifestyle coding, E - core machinery coding. * of occurrences in related species. [min,max] - minimal and maximal number of species within a gene group (O, M, E) and conservation group.
(EPS)

Figure S5 **Shannon entropy of the genome versus the maximal possible growth rate.** The 16 *in silico* strains are shown with their predicted growth rates in glucose minimal medium/aerobic condition (GlcAer), glucose minimal medium/anaerobic condition (GlcAnaer), glycerol minimal medium/aerobic condition (Glyc) and lactate minimal medium, aerobic conditions (Lac). Eq. strains = equilibrated strains. WT = wildtype.
(EPS)

Figure S6
**Reduced cost results are shown for the biased strains in the four defined growth conditions.** Increasing the flux rate through the transcription reaction of the tRNA transcription units (e.g., tscr_iniTU00518_stab encoding 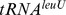) by 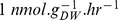 would increase the growth rate by 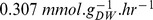 (B1). The RCs are given in 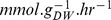. Inset: changes of major leucine codon in the CUB perturbed ME-matrix. tRNA recognizing the codon is given in parenthesis. Leu1 and Leu2 are generic tRNA species representing multiple leucyl-tRNA species, see Table S2.(EPS)

Table S1 **Summary of genomes included in the gene conservation analysis.** Open Reading Frames (ORFs).
(PDF)

Table S2 textbfCodons recognition by tRNA in the ME-matrix.
(PDF)
