# Supplementary material for: Multiscale Modeling of Metabolism and Macromolecular Synthesis in E. coli and Its Application to the Evolution of Codon Usage
Source: PLoS One. 2012 Sep 28;7(9):e45635. doi: 10.1371/journal.pone.0045635 (PMC3461016; doi:10.1371/journal.pone.0045635)
Supplement: Table S1 — Summary of genomes included in the gene conservation analysis. Open Reading Frames (ORFs). (PDF) [file pone.0045635.s007.pdf]

Supplementary Table S1

Summary of genomes included in the gene conservation analysis. Part I. Open Reading Frames (ORFs).

	NAME	ORFs	genes mapped (in %)
N.	ENTEROBACTERS		
1	0127 H6 E2348 69	4653	54
2	536	4620	55
3	APEC O1	4851	51
4	Buchnera aphidicola	507	92
5	Buchnera aphidicola 5A Acyrthosiphon pisum	555	93
6	Buchnera aphidicola Cc Cinara cedri	357	86
7	Buchnera aphidicola Sg	546	92
8	Buchnera aphidicola Tuc7	553	93
9	Buchnera sp	574	92
10	Candidatus Blochmannia floridanus	583	92
11	Candidatus Blochmannia pennsylvanicus BPEN	610	92
12	CFT073	5339	48
13	Citrobacter koseri ATCC BAA-895	5008	47
14	C ATCC 8739 NC	4200	64
15	E24377A	4991	53
16	Enterobacter sakazakii ATCC BAA-894	4420	48
17	Erwinia carotovora atroseptica SCRI1043	4472	45
18	Erwinia tasmaniensis	3622	50
19	HS	4378	60
20	Klebsiella pneumoniae 342	5768	40
21	Klebsiella pneumoniae MGH 78578	5187	45
22	K 12 substr DH10B	4126	65
23	O157H7 EDL933	5411	48
24	O157H7 NC 002128 all	5318	49
25	O157 H7 EC4115	5477	47
26	Paratyphi A AKU 12601	4078	56
27	Paratyphi B SPB7	5592	42
28	Photorhabdus luminescens	4683	37
29	Proteus mirabilis NC	3662	49
30	Salmonella Agona SL483 faa	4614	50
31	Salmonella Enteritidis P125109	4206	56
32	Salmonella Gallinarum 287 91	3965	58
33	Salmonella Heidelberg SL476	4779	49
34	Salmonella Newport SL254	4805	49
35	Salmonella typhimurium LT2	4525	52
36	Salmonella typhi NC	4758	48
37	Salmonella Typhi Ty2	4318	53
38	Schwarzengrund CVM19633	4627	50
39	SE11	5002	53
40	Serratia proteamaculans 568	4942	45
41	Shigella boydii CDC 3083 94	4557	50
42	Shigella boydii Sb227	4283	58

Summary of genomes included in the gene conservation analysis. Part II

	NAME	ORFs	genes mapped (in %)
43	Shigella dysenteriae	4503	48
44	Shigella flexneri 2a 2457T	4061	60
45	Shigella flexneri 2a NC 004337	4440	54
46	Shigella flexneri 5 8401	4115	60
47	Shigella sonnei Ss046	4471	59
48	SMS 3 5	4913	54
49	Sodalis glossinidius morsitans	2516	55
50	UTI89 vs eco 2 query.ko	5021	51
51	W3110	4226	65
52	Wigglesworthia brevipalpis	617	90
53	Yersinia enterocolitica 8081	4051	51
54	Yersinia pestis Angola	4040	46
55	Yersinia pestis Antiqua	4364	45
56	Yersinia pestis biovar Microtus 91001	4138	47
57	Yersinia pestis CO92	4066	47
58	Yersinia pestis KIM	4202	46
59	Yersinia pestis Nepal516	4094	48
60	Yersinia pestis Pestoides F	4069	48
61	Yersinia pseudotuberculosis IP32953	4038	49
62	Yersinia pseudotuberculosis IP 31758	4324	46
63	Yersinia pseudotuberculosis PB1	4237	47
64	Yersinia pseudotuberculosis YPIII	4192	47
65	Yersinia pseudotuberculosis IP32953 '	4038	49
	NON-ENTEROBACTERS		
66	Acinetobacter baumannii AYE NC 010410 all	3712	36
67	Acinetobacter sp ADP1 NC 005966	3307	40
68	Bacillus subtilis NC 000964	4105	32
69	Clostridium beijerinckii NCIMB 8052 NC 009617	5020	23
70	Clostridium botulinum E3 Alaska E43 NC 010723	3256	26
71	Clostridium thermocellum ATCC 27405 NC 009012	3189	24
72	Corynebacterium glutamicum ATCC 13032 Bielefeld NC 006958	3057	27
73	Corynebacterium glutamicum ATCC 13032 Kitasato NC 003450	2993	33
74	Geobacillus thermodenitrificans NG80-2 NC 009328 all	3445	29
75	Geobacter lovleyi SZ NC 010814 all	3685	27
76	Geobacter uraniumreducens Rf4 NC 009483	4357	24
77	Haemophilus influenzae 86 028NP NC 007146	1792	62
78	Haemophilus influenzae NC 000907	1657	68
79	Helicobacter pylori G27 NC 011333 all	1504	37
80	Helicobacter pylori HPAG1 NC 008086 all	1544	38
81	Lactobacillus acidophilus NCFM NC 006814	1862	31
82	Lactobacillus delbrueckii bulgaricus NC 008054	1562	33
83	Lactococcus lactis cremoris MG1363 NC 009004	2434	30
84	Lactococcus lactis cremoris SK11 NC 008527 all	2504	28
85	Mannheimia succiniciproducens MBEL55E NC 006300	2369	55
86	Mycobacterium tuberculosis H37Ra NC 009525	4034	20

Summary of genomes included in the gene conservation analysis. Part III

	NAME	ORFs	genes mapped (in %)
87	Mycoplasma agalactiae PG2 NC 009497	742	32
88	Mycoplasma capricolum ATCC 27343 NC 007633	812	33
89	Nautilia profundicola AmH NC 012115	1730	39
90	Neisseria gonorrhoeae FA 1090 NC 002946	2002	43
91	Neisseria meningitidis 053442 NC 010120	2020	44
92	Porphyromonas gingivalis ATCC 33277 NC 010729	2090	30
93	Pseudomonas aeruginosa PA7 NC 009656	6286	26
94	Pseudomonas fluorescens Pf-5 NC 004129	6138	27
95	Pseudomonas mendocina ymp NC 009439	4594	32
96	Pseudomonas putida GB 1 NC 010322	5409	30
97	Pseudomonas stutzeri A1501 NC 009434	4128	35
98	Rhizobium etli CFN 42 NC 007766 all	5963	21
99	Rhizobium leguminosarum bv viciae 3841 NC 008384 all	7143	19
100	Staphylococcus aureus Newman NC 009641	2614	31
101	Staphylothermus marinus F1 NC 009033	1570	15
102	Streptomyces griseus NBRC 13350 NC 010572	7136	15
103	Synechococcus PCC 7002 NC 010480 all	3186	25
104	Thermotoga petrophila RKU-1 NC 009486	1785	37
105	Thermotoga RQ2 NC 011978	1937	35
