# Supplementary material for: Multiscale Modeling of Metabolism and Macromolecular Synthesis in E. coli and Its Application to the Evolution of Codon Usage
Source: PLoS One. 2012 Sep 28;7(9):e45635. doi: 10.1371/journal.pone.0045635 (PMC3461016; doi:10.1371/journal.pone.0045635)
Supplement: Table S2 — textbfCodons recognition by tRNA in the ME-matrix. (PDF) [file pone.0045635.s008.pdf]

Supplementary Table S2

Codons recognition by tRNA in the ME-matrix. Part I.

	generic tRNA	codon	Amino acid
alaT, alaU, alaV	ala1-tRNA	gct	ala-L
alaT, alaU, alaV	ala1-tRNA	gca	ala-L
alaT, alaU, alaV	ala1-tRNA	gcg	ala-L
alaW, alaX	ala2-tRNA	gcc	ala-L
argQ, argV, argY, argZ	arg1-tRNA	cgt	arg-L
argQ, argV, argY, argZ	arg1-tRNA	cgc	arg-L
argQ, argV, argY, argZ	arg1-tRNA	cga	arg-L
	argU-tRNA	aga	arg-L
	argW-tRNA	agg	arg-L
	argX-tRNA	cgg	arg-L
asnT, asnU, asnV, ansW	asn1-tRNA	aac	asn-L
asnT, asnU, asnV, ansW	asn1-tRNA	aat	asn-L
aspT, aspU, aspV	asp1-tRNA	gac	asp-L
aspT, aspU, aspV	asp1-tRNA	gat	asp-L
	cysT-tRNA	tgc	cys-L
	cysT-tRNA	tgt	cys-L
glnU, glnW	gln1-tRNA	cag	gln-L
glnV, glnX	gln2-tRNA	caa	gln-L
gltT, gltU, gltV, gltW	glu1-tRNA	gaa	glu-L
gltT, gltU, gltV, gltW	glu1-tRNA	gag	glu-L
glyV, glyW, glyX, glyY	gly1-tRNA	ggc	gly
glyV, glyW, glyX, glyY	gly1-tRNA	ggt	gly
	glyT-tRNA	gga	gly
	glyU-tRNA	ggg	gly
	hisR-tRNA	cac	his-L
	hisR-tRNA	cat	his-L
ileT, ileU, ileV	ile1-tRNA	atc	ile-L
ileT, ileU, ileV	ile1-tRNA	att	ile-L
ileX, ileY	ile2-tRNA	ata	ile-L
leuP, leuQ, leuT, leuV, leuW	leu1-tRNA	ctg	leu-L
	leuU-tRNA	ctc	leu-L

Codons recognition by tRNA in the ME-matrix. Part II.

	generic tRNA	codon	Amino acid
	leuU-tRNA	ctt	leu-L
	leuW-tRNA	cta	leu-L
leuX, leuZ	leu2-tRNA	ttg	leu-L
	leuZ-tRNA	tta	leu-L
lysQ, lysT, lysV, lysW, lysY, lysZ	lys1-tRNA	aaa	lys-L
lysQ, lysT, lysV, lysW, lysY, lysZ	lys1-tRNA	aag	lys-L
metT, metU	met1-tRNA	atg	met-L
pheU, pheV	phe1-tRNA	ttc	phe-L
pheU, pheV	phe1-tRNA	ttt	phe-L
proK, proM	pro1-tRNA	ccg	pro-L
	proL-tRNA	ccc	pro-L
proL, proM	pro2-tRNA	cct	pro-L
	proM-tRNA	cca	pro-L
serW, serX	ser1-tRNA	tcc	ser-L
serT, serW, serX	ser2-tRNA	tct	ser-L
	serT-tRNA	tca	ser-L
serT, serU	ser3-tRNA	tcg	ser-L
	serV-tRNA	agc	ser-L
	serV-tRNA	agt	ser-L
thrT, thrV	thr1-tRNA	acc	thr-L
thrT, thrU, thrV	thr2-tRNA	act	thr-L
	thrU-tRNA	aca	thr-L
thrU, thrW	thr3-tRNA	acg	thr-L
	trpT-tRNA	tgg	trp-L
tyrT, tyrU, tyrV	tyr1-tRNA	tac	tyr-L
tyrT, tyrU, tyrV	tyr1-tRNA	tat	tyr-L
valT, valU,valX, valY, valZ	val1-tRNA	gta	val-L
valT, valU,valX, valY, valZ	val1-tRNA	gtg	val-L
valV, valW	val2-tRNA	gtc	val-L
valT, valU, valW, valX, valY, valZ	val3-tRNA	gtt	val-L
metV, metW, metY, metZ	fmet-tRNA	atg	start
